# Indications of Brain Computed Tomography Scan in Children Younger Than 3 Years of Age with Minor Head Trauma

**DOI:** 10.1155/2014/248967

**Published:** 2014-03-02

**Authors:** İsmail Gülşen, Hakan Ak, Sevdegül Karadaş, İsmail Demır, Mehmet Deniz Bulut, Soner Yaycioğlu

**Affiliations:** ^1^Yüzüncü Yıl University, School of Medicine, Department of Neurosurgery, Van, Turkey; ^2^Bozok University, School of Medicine, Department of Neurosurgery, Yozgat, Turkey; ^3^Yüzüncü Yıl University, School of Medicine, Department of Emergency Medicine, Van, Turkey; ^4^Van Regional Training and Teaching Hospital, Clinic of Neurosurgery, Van, Turkey; ^5^Yüzüncü Yıl University, School of Medicine, Department of Radiology, Van, Turkey; ^6^Adnan Menderes University, School of Medicine, Department of Neurosurgery, Aydın, Turkey

## Abstract

*Objective*. To investigate the indications to receive brain computed tomography (CT) scan and to define the pathological findings in children younger than three years of age with minor head trauma in emergency departments. *Methods*. In this study, hospital case notes of 1350 children attending the emergency department of Bitlis State Hospital between January 2011 and June 2013 were retrospectively reviewed. 508 children under 3 years of age with minor head trauma were included in this study. We also asked 37 physicians about the indications for requiring CT in these children. *Results*. This study included 508 children, 233 (45,9%) of whom were female and 275 were male. In 476 (93,7%) children, the brain CT was completely normal. 89,2% of physicians asked in the emergency department during that time interval reported that they requested CT scan to protect themselves against malpractice litigation. *Conclusion*. In infants and children with minor head trauma, most CT scans were unnecessary and the fear of malpractice litigation of physicians was the most common reason for requesting a CT.

## 1. Introduction

Head trauma is the leading cause of mortality and morbidity in children [1]. Minor head trauma (MHT) constitutes a significant proportion of head injuries in children, about 90% [1, 2]. The prevalence, morbidity, and costs related to it make it an important health problem [2]. However, the incidence of intracranial pathology in MHT is about 3–5% [1]. As a general rule, in pediatric trauma patients with a Glasgow Coma Scale (GCS) less than 13, focal neurological deficits, and deteriorating consciousness should receive CT scan. However, for children with milder head injury, there is no clear consensus about requesting CT [3].

Most of the children with minor head trauma attend the emergency department nonsymptomatically or with minimal symptoms. Neurological examination is difficult in children, especially in newborns, infants (between one month and 12 months), and those under 3 years of age. Also, concern of the parents for their children and fear of malpractice litigation may force the physicians to request radiological imaging, especially the CT. The rate of requesting CT scans in children with MHT is between 5 and 50% [1]. The literature does not have sufficient data about the need for CT in this age group attending with MHT.

In this present study, we retrospectively evaluated CT findings in infants and in children under 3 years of age with MHT and the indications for their request.

## 2. Methods

In this study, hospital case notes of 1350 children attending the emergency department of Bitlis State Hospital between January 2011 and June 2013 were retrospectively reviewed. 508 children younger than 3 years of age with MHT (Modified GCS 14 or GCS 15) were included in the study. Patients with insufficient data or those considered as child abuse were excluded. Information about age, gender, type of trauma, physical and neurological examination findings, CT result, and last status (discharge, hospitalization, or referral) was obtained from patients' hospital case notes.

37 physicians who worked during this time were asked about their reasons for requesting the CT scan. These roughly divided into four groups: protecting themselves against malpractice litigation, request of the family, not knowing indications for requesting the scan, and not wanting to follow up the patient in the emergency room.

## 3. Statistical Analysis

The statistical analyses were performed using software (SPSS 18.0). Parametric values were given as mean ± standard deviation, and nonparametric values were given as a percentage. To compare parametric continuous variables, student's *t*-test or analysis of variance was used; to compare nonparametric continuous variables, the Mann-Whitney *U* test or the Kruskal-Wallis test was used. Categorical data were compared by Chi-square distribution. Two-tailed *P* values of less than 0.05 were considered to indicate statistical significance.

## 4. Results

508 children younger than 3 years of age with minor head trauma were included in the study. 233 (45,9%) of them were female and 275 (54,1%) were male. 157 of them (30,9%) were younger than one year of age. 216 (42,5%) and 135 (26,6%) of them were between one and two years of age and two and three years of age, respectively. 89,5% of them were totally asymptomatic. In others presenting symptoms were vomiting in 38 (7,5%) and loss of consciousness for less than 5 seconds in 23 (4,5%) children. In 8 children, the presenting symptom was both vomiting and loss of consciousness.

Physical examination was totally normal except 40 (7,9%) cases. Brain CT scan revealed no pathological findings, cephalohematoma, linear fracture, cerebral contusion, epidural hematoma, and subdural hematoma in 476 (93,7%), 3 (0,6%), 18 (3,6%), 5 (1%), 2 (0,4%), and 4 (0,8%) of children, respectively. None of these children with pathological CT findings required operation. 476 (93,7%) of them had been discharged about 3 hours after trauma. The 18 children with linear fracture had been followed in the emergency service for about 24 hours. 14 children with cerebral contusion, epidural hematoma, and subdural hematomas were hospitalized for clinical followup for about 48 hours in the neurosurgery clinic.

There was a statistically significant relationship between vomiting and pathological physical examination findings (*P* = 0,001; *R* = 0,945). A similar relationship between loss of consciousness and pathological physical examination findings was detected (*P* = 0,001;  *R* = 0,253).

There were significant correlations between vomiting and pathological CT findings (*P* = 0,001;  *R* = 0,542), loss of consciousness and pathological CT findings (*P* = 0,001;  *R* = 0,450), and pathological physical examination findings and pathological CT findings (*P* = 0,001;   *R* = 0,556).

The physicians and reasons of requesting CT divided into groups. Physicians divided into five groups. These are neurosurgeons (group 1, *n* = 2), specialists in emergency medicine (group 2, *n* = 2), specialists in internal sciences (group 3, *n* = 9), specialists in surgical sciences (group 4, *n* = 12), and general practitioners (group 5, *n* = 12). Answers were grouped as protecting themselves against malpractice litigation, request of the family, not knowing indications of requesting CT scan, and not wanting to follow up the patient in the emergency room. 33 (89,2%) physicians (groups 3, 4, and 5) stated that they requested CT to protect themselves against malpractice litigation. 21 (56,8%) (groups 3 and 5) physicians stated their indication as the request of the family. 24 (64,9%) (groups 4 and 5) physicians answered this question as not wanting to follow up the child in the emergency room. 14 (37,8%) physicians (6 physicians in group 3, 4 physicians in group 4, and 4 physicians in group 5) stated their answers as not being sure about indications of CT scanning in head trauma (Table 1).

Physicians in group 1 requested 20 CTs and there was a pathological CT finding on all of them. Physicians in group 2 requested 33 CTs and there was a pathological CT finding on 12 of them. Physicians requested 170, 100, and 185 CT scans in groups 3, 4, and 5, respectively.

## 5. Discussion 

Head injury is common in pediatric population [1, 4]. In the presence of signs and symptoms of severe head injury like decreased GCS score, focal deficit/s, and deteriorating consciousness, the diagnosis of this pathology is relatively easy [1]. However, in MHT, which constitutes about 90% of childhood head trauma, the management of these patients is not so easy and there are some controversial points especially in appropriate diagnostic assessment [2, 3, 5]. Despite its very high prevalence, only 3–5% of children with MHT have traumatic brain injury and only less than 1% of them need emergent neurosurgical intervention [6]. Previous reports from different countries about pediatric minor head trauma indicated various results in clinical practice and examination of hospitalization rates [5].

Although in adult patients with MHT clinical decision rules for requesting CT have been well documented, performing a similar decision rule in children with MHT has two important difficulties [7, 8]. The first is difficulty in developing an appropriate data set, with adequate representation of all age groups and an adequate number of cases of traumatic brain injury. The second one is defining clinical variables that accurately predict traumatic brain injury in childhood [8].

Many of the previous studies about pediatric MHT in the literature have been retrospective from a single institution and have included a small number of patients. Also, these studies have included a limited number of infants and very young children [8]. Definite indications for CT scanning after a head trauma are a deteriorating clinical course, focal neurologic deficit, abnormal mental status, evidence of a skull fracture, and the presence of coagulopathy. Also, until additional evidence is available, loss of consciousness and persistent vomiting (more than 3) should be taken into consideration [9]. In a meta-analysis of 16 studies including 22420 children with MHT, Dunning et al. reported that the presence of skull fracture, focal neurologic signs, loss of consciousness, and GCS less than 15 was statistically associated with traumatic brain injury, whereas headache and vomiting were not. They also stated that the presence of seizures showed a trend toward an association with traumatic brain injury, but not a statistically significant association [10]. In a case control study, da Dalt et al. reported a similar conclusion that vomiting after an MHT was significantly related to personal or familial predisposition to vomit rather than to the presence of intracranial lesions [11]. However, in our study we observed strong relationship between vomiting and loss of consciousness and pathological CT findings.

There are two main problems concerning CT scans in children: most importantly the exposure to radiation and secondarily the cost. As we know, children, especially younger than 2 years of age, are considerably more sensitive to radiation than adults due to rapidly dividing cells associated with growth [12, 13]. Since children have longer life expectancies, they have more opportunity to express radiation damage, particularly cancer [12]. Additionally, we learned that a single head CT exposes children to 200–600 times as much radiation as typical posteroanterior and lateral chest radiographs [14].

According to the results of the present study, CT scans were requested unnecessarily in 93,7% of our patients. In fact, the aim of our study was to highlight this subject. For this purpose, we asked 37 physicians about the indications for requiring CT scans in these children to investigate the possible causes of high scanning rates. Our results clearly showed that the fear of malpractice litigation is the most important factor. Studdert et al. reported a similar conclusion in their study [15]. It was reported that, only in 2010, 9497 medical practice claims were filed in the United States and totally $3,177,305,000 were paid for these claims (average $334,559) [16]. Average payment constitutes about 5–10 times of the annual income of a physician in Turkey. The second most important factor is the request of the family. In our opinion, the request of the family should not have any effect in our daily practice; however, again the fear of malpractice litigation secondarily changes our practice. 14 physicians (37,8%) in our study stated that they were not sure about definite indications for brain CT in these children. Our results also indicate that neurosurgeons and specialists in emergency medicine had a high rate of detecting a pathological finding on CT. However, these physicians might miss some cases with pathological findings.

## 6. Conclusions 

There is a need for further prospective, multicentered studies with a large number of patients to make decision rules especially for children in this age group.

The fear of malpractice litigation should be reduced by various measures which will protect physicians such as robust departmental guidelines.

In emergency departments, the number of specialists or qualified and certificated staff should be increased. Other physicians working in the emergency services should be trained intermittently in these issues.

*Limitations.* There are two main limitations in our study. These are the retrospective nature of the study and the fact that it is single centered.

## Figures and Tables

**Table 1:** Physician groups and their reasons for requesting CT scan.

Group	Malpractice litigation	Request of the family	Not knowing indications	Not wanting to follow up
G1 (*n* = 2)	−	−	−	−
G2 (*n* = 2)	−	−	−	−
G3 (*n* = 9)	+	+	−	+ (6 of them)
G4 (*n* = 12)	+	−	+	+ (4 of them)
G5 (*n* = 12)	+	+	+	+ (4 of them)

## References

[1] Quayle KS, Jaffe DM, Kuppermann N (1997). Diagnostic testing for acute head injury in children: when are head computed tomography and skull radiographs indicated?. *Pediatrics*.

[2] Savitsky EA, Votey SR (2000). Current controversies in the management of minor pediatric head injuries. *The American Journal of Emergency Medicine*.

[3] Simon B, Letourneau P, Vitorino E, McCall J (2001). Pediatric minor head trauma: indications for computed tomographic scanning revisited. *Journal of Trauma*.

[4] Schutzman SA, Greenes DS (2001). Pediatric minor head trauma. *Annals of Emergency Medicine*.

[5] Homer CJ, Kleinman L (1999). Technical report: minor head injury in children. *Pediatrics*.

[6] Özsaraç M, Karçıoğlu Ö, Topaçoğlu H (2009). Clinical indicators of traumatic brain injury and skull fracture in pediatric head trauma patients. *Turkish Journal of Emergency Medicine*.

[7] Haydel MJ, Preston CA, Mills TJ, Luber S, Blaudeau E, DeBlieux PMC (2000). Indications for computed tomography in patients with minor head injury. *The New England Journal of Medicine*.

[8] Greenes DS (2003). Decision making in pediatric minor head trauma. *Annals of Emergency Medicine*.

[9] Gordon KE (2006). Pediatric minor traumatic brain injury. *Seminars in Pediatric Neurology*.

[10] Dunning J, Batchelor J, Stratford-Smith P (2004). A meta-analysis of variables that predict significant intracranial injury in minor head trauma. *Archives of Disease in Childhood*.

[11] da Dalt L, Andreola B, Facchin P, Gregolin M, Vianello A, Battistella PA (2007). Characteristics of children with vomiting after minor head trauma: a case-control study. *The Journal of Pediatrics*.

[12] Schnadower D, Vazquez H, Lee J, Dayan P, Roskind CG (2007). Controversies in the evaluation and management of minor blunt head trauma in children. *Current Opinion in Pediatrics*.

[13] Kuppermann N, Holmes JF, Dayan PS (2009). Identification of children at very low risk of clinically-important brain injuries after head trauma: a prospective cohort study. *The Lancet*.

[14] National Cancer Institute (2002). *Radiation Risks and Pediatric Computed Tomography (CT): A Guide for Healthcare Providers*.

[15] Studdert DM, Mello MM, Sage WM (2005). Defensive medicine among high-risk specialist physicians in a volatile malpractice environment. *The Journal of the American Medical Association*.

[16] Drukteinis DA, O’Keefe K, Sanson T, Orban D (2014). Preparing emergency physicians for malpractice litigation: a joint emergency medicine residency-law school mock trial competition. *The Journal of Emergency Medicine*.

